# Grand Challenges in Adolescent Sexual and Reproductive Health

**DOI:** 10.3389/frph.2020.00002

**Published:** 2020-06-18

**Authors:** Supriya Dinesh Mehta, Janet Seeley

**Affiliations:** ^1^School of Public Health, University of Illinois at Chicago, Chicago, IL, United States; ^2^London School of Hygiene and Tropical Medicine, London, United Kingdom

**Keywords:** adolescents, sexual health, risks & opportunities, reproductive health, young people

At the International Conference on Population and Development in Cairo in 1994, the sexual and reproductive health needs of adolescents were highlighted, with calls for countries to address both the educational and service needs of young people (1, 2) while securing the reproductive rights of women and girls (3). Much progress has been made since that time, with heightened awareness that sustaining sexual health in adolescence plays an essential part in reproductive health and wellbeing in later life (4, 5), but challenges remain in ensuring access to reproductive health care and education and addressing entrenched gender norms which continue to affect both young women and young men as they manage the transition to adulthood (6).

Over the past 30 years the HIV epidemic has played a role in shaping the agenda on adolescent health, particularly in sub-Saharan Africa (7, 8). Considerable attention has been paid, with reason, to the prevention of HIV infection among young women, given high levels of HIV-incidence in this population in, for example, southern Africa (9). The narrative around protecting young women from HIV infection has influenced the framing of interventions (10) and assumptions around risk and vulnerability being different for adolescent boys and girls (11). Given gender disparities in power which place girls and women at a disadvantage in their relationships with men in many settings, and concerns around the impact of early marriage and unintended pregnancies, as well as HIV acquisition for adolescent girls (12), the greater attention paid to the sexual and reproductive health of girls and young women is justified. However, the narrative of protection of girls and young women risks marginalizing boys and young men, or at the least viewing their sexual health as consisting of sexual desires to control (13) rather than acknowledging their broader reproductive health education and service access needs. This also underlines the continuing importance of addressing the gendered norms that perpetuate expectations of particular forms of masculine behavior by both young men and young women (14).

Adolescence is generally defined as being the time between puberty and reaching adult independence (15), with the age range included most commonly being from 10 to 19 years (16). Puberty is considered a key element in human development into adulthood (17, 18), yet this marker of physical maturity is occurring at earlier ages in many settings, accompanied by an earlier onset of sexual attention, sexual thoughts, and experimentation (19). Understanding and managing puberty in childhood, well-before what has been considered ‘adolescence,' requires urgent attention. For girls, this management is often focused on their experience of menarche. Menstrual health is an area which has focused attention on the reproductive health needs of girls and young women (20), with a growing body of literature documenting successful interventions in schools (21–23). There is also a recognition of the importance of support for menstruation support and management which includes boys and young men to heighten their awareness and sensitivity to the issues (24), so that their attitudes and behavior do not promote the exclusion from school of girls experiencing their periods. The inclusion in policy discussions and interventions that the reproductive health concerns of girls and boys, young women and young men, are interlinked beyond males being viewed as sexually dominant, helps in the recognition of the sexual health uncertainties that boys as well as girls face in adolescence and beyond.

Sexually transmitted Infections (STIs) are another major health concern for adolescents. Globally, adolescent girls and young women account for at least one-third of the 357 million curable STIs occurring each year (25–27). STIs are an important focus due to their long-term reproductive health consequences related to fertility and pregnancy outcomes (28, 29) and cervical cancer (30). A broad range of socioecological factors create unique disadvantage and vulnerability to STIs for adolescents (31), and this context leads to co-occurring risks, including difficulty accessing contraception and safe abortion, and early pregnancy and parenthood. Multiple levels from the socioecological sphere are unique to adolescents or felt more keenly by adolescents, such as psychological aspects (e.g., cognitive development, mental health), interactions with peers and family, socio-economic status and lack of economic autonomy, health care policies, social media, cultural perspectives, and multiple dimensions of discrimination. These socioecological factors contribute to multiple syndemics among adolescents: mental health concerns, interpersonal violence, poverty and lack of educational and economic opportunity, and substance use (32–35). Generating and disseminating evidence to understand multiple adolescent SRH outcomes simultaneously with regard to the shared ecosystem in which risks occur can optimize development of more effective and multiple purpose interventions. Some of the barriers to generating socioecological system data include silo-ing of disciplines, coordination of policy and funding across multiple sectors, and the common emphasis on single level or single exposure studies (35). Simultaneously addressing multiple levels of the socioecological sphere may lead to more effective and sustainable interventions (31, 36), and can even be incorporated in study processes to improve recruitment and retention (37), supporting more successful scaling.

While the past decade has seen progress in rights and academic scholarship for lesbian, gay, bisexual transgender, queer or questioning (LGBTQ) persons, homosexuality/transgender remains illegal or highly stigmatized in many countries (38), and children and adolescents who are LGBTQ face substantial discrimination, harassment, and violence (39). While separate concepts, sexual orientation, gender identity, and intersex are often linked by common issues of safety and promoting and protecting sexual health. Promoting human rights to condemn abuses related to sexual orientation or gender identity and to protect and provide supportive services for LGBTQ youth are a priority (39). However, data are limited on how best to develop and implement practices and policies that support LGBTQ youth, and much originates in North America or Europe (40) with underrepresentation in countries with the greatest stigma. The challenges of adolescent health research can be amplified by: difficulty measuring sexual orientation, identity, attraction, and behavior along developmental trajectories (40), inadvertent risk of “outing” to parents, peers, or community (41), and increased sensitivity and stigmatization of LGBTQ research (42).

Conducting adolescent sexual and reproductive health research is challenging, as there are complex ethical considerations for protecting confidentiality and privacy, obtaining informed consent, and addressing vulnerabilities (43–45). Adolescent SRH may also face political challenges and limited funding opportunities (46, 47). As a result, adolescent SRH research can be limited in scope, scale, methodologic rigor, and explanatory power, with resultant limitations for generalizability, reproducibility, and dissemination. Despite this, research done under these limitations may still have merit, but it may be modest and fail to find an audience or contribute to progress in the field. While greater investment in adolescent SRH research is needed, in the current context, we encourage high quality and innovative studies—as well as research which yields negative/unexpected results, pilot studies, hypotheses, and concepts—to accelerate discovery in adolescent SRH.

Given the many gaps and challenges in understanding determinants and barriers of adolescent SRH and how to optimize translation of existing knowledge, innovation is a critical component of the *Global Strategy* for generating evidence, prioritizing local needs and capacities, and supporting active engagement across sectors to improve adolescent health outcomes (48).

Below, we outline some areas of innovation and investigations that have promise for shifting paradigms and pioneering breakthroughs in adolescent SRH:

1. **Improving acceptability and reducing potential harms in obtaining informed consent**. As Fisher and Mustanski note, adolescent SRH is particularly susceptible to overestimation of research risk (42). SRH research involving LGBTQ youth is even more likely to be “classified as greater than minimal risk due to unsubstantiated assumptions” that answering questions about sexual practices and harms will have adverse outcomes (42). Guardian permission poses additional challenge. Knopf et al. summarize their proposed research on consent methods and processes for adolescent HIV research which can generate guidance for investigators, regulatory boards, and donors (45). This is critically needed because minors are often excluded from clinical trials, especially from biomedical HIV interventions, due to ethical complexities, generally involving parental consent. Even when included in research involving sexual behaviors, adolescents can be unwilling to enroll due to potential social harms. We need studies of new consent methods and ethical review processes for multiple types of studies designs, settings, and content related to adolescent SRH, across global sociocultural contexts. This is especially important in youth for whom there is insufficient knowledge on SRH: adolescents under age 15, youth in vulnerable situations (e.g., refugees, street youth), LGBTQ youth, and males (42, 45, 49).
2. **Novel approaches to facilitate and expand participation, measure of multiple adolescent SRH risks and outcomes, and translation and scalability**. Community-engaged participatory research can lead to new questions, improved messaging, increased recruitment of diverse people, and development of new measurement tools (50). This type of approach may be even more relevant for adolescents, as they become more independent and experience profound changes in identity and socialization. Youth-led and youth-engaged approaches enable adolescents to play a central role in the problem definition, and development, content, and design of research studies and interventions, with special relevance for giving voice to marginalized groups. Ozer and Piatt describe the underlying theories and provide examples for application in research design and methods, and analysis; they highlight the need for assessing validity of findings from such approaches, and developing models with broad initiatives and scale (51).
3. **Improve accessibility and relevance of resources and interventions with technological solutions**, such as peer-to-peer video conferencing (36), or online interventions through applications via Web or mobile technology (52). Bacchus et al. summarize other opportunities—such as online STI testing, online contraception ordering, and distribution of abortifacient pills—that can overcome geographic or social barriers (53). However, such research for adolescents is lacking. We need research that demonstrates creative uses of technology for adolescents: infrastructure and implementation processes and needs, factors that optimize acceptability, safety, and utility, measurement of positive effects for adolescent SRH (e.g., puberty and health sexuality education, improved access to SRH care and services, social support), and methods to identify and mitigate negative effects (e.g., increased exposure to risk, unintended adverse outcomes).

## Concluding Remarks

Over the past 30 years, there have been significant advances in adolescent SRH—increasing delays in marriage and first childbirth, and gains in education and economic development. Nevertheless, adolescents face a rapidly changing world. The disruptions due to climate change, conflict, and pandemics are increasing in frequency, severity, and impact. We must not lose the momentum of improvements in adolescent SRH. This new Frontiers section on Adolescent Reproductive Health aims to present to its readers advances in existing and emerging areas of research, with an eye to the broader context of adolescent SRH, and welcomes bold, innovative ideas to develop and invigorate the field. We are excited to shepherd a diverse and inclusive range of views and perspectives related to adolescent sexual and reproductive health.

## Author Contributions

All authors listed have made a substantial, direct and intellectual contribution to the work, and approved it for publication.

## Conflict of Interest

The authors declare that the research was conducted in the absence of any commercial or financial relationships that could be construed as a potential conflict of interest.
